# Systemic Lupus Erythematosus-Associated Myelitis Mimicking Spinal Glioblastoma: A Case Report

**DOI:** 10.7759/cureus.102763

**Published:** 2026-02-01

**Authors:** Yuki Sakaeyama, Shuhei Kubota, Hiroshi Takahashi, Eisuke Tanaka, Nobuo Sugo

**Affiliations:** 1 Department of Neurosurgery, Toho University, Tokyo, JPN; 2 Department of Orthopaedic Surgery, Toho University, Tokyo, JPN; 3 Division of Rheumatology, Department of Internal Medicine, Toho University, Tokyo, JPN

**Keywords:** glucocorticoid, intramedullary spinal tumor, myelitis, spinal glioblastoma, systemic lupus erythematosus

## Abstract

Systemic lupus erythematosus (SLE)-associated myelitis is an uncommon but potentially disabling neurological manifestation that can present with radiological features resembling other intramedullary spinal cord disorders. Imaging findings may overlap with demyelinating diseases or intramedullary tumors such as spinal glioblastoma, which can lead to diagnostic uncertainty and consideration of invasive procedures. Early recognition is important because prompt initiation of glucocorticoid (GC) and immunosuppressive therapy may result in neurological improvement.

A 48-year-old woman with a long-standing history of SLE presented with progressive numbness and mild weakness of the lower extremities. Spinal MRI demonstrated an intramedullary lesion at the Th1-2 level with T2 hyperintensity and heterogeneous gadolinium enhancement, initially raising suspicion for spinal glioblastoma. Cerebrospinal fluid analysis revealed mild pleocytosis without oligoclonal bands, and both aquaporin-4 and myelin oligodendrocyte glycoprotein antibodies were negative. Given the possibility of inflammatory myelitis, GC therapy was initiated. Her sensory symptoms improved within several days, and a follow-up MRI performed one week later showed a marked reduction in lesion size and contrast enhancement. Surgical intervention was therefore deferred. The patient has remained relapse-free for 20 months under a gradual GC taper.

This case demonstrates that SLE-associated myelitis can closely mimic spinal glioblastoma on MRI. Responsiveness to GC therapy and short-interval follow-up imaging can provide important diagnostic clues and help avoid unnecessary surgical procedures. Autoimmune myelitis should be considered in the differential diagnosis of intramedullary spinal cord lesions.

## Introduction

Systemic lupus erythematosus (SLE) is an autoimmune disorder characterized by diverse neurological manifestations, collectively referred to as neuropsychiatric SLE. Among these, SLE-associated myelitis is an uncommon but potentially disabling complication, occurring in approximately 1-2% of SLE patients and often presenting with rapidly progressive neurological deterioration requiring urgent treatment [1]. Clinically, it typically manifests with an acute or subacute onset of sensory disturbance, motor weakness, and occasionally sphincter dysfunction.

Recent cohort studies have demonstrated that SLE-associated myelitis may present with longitudinally extensive spinal lesions, variable autoantibody profiles, and heterogeneous clinical outcomes, highlighting the complexity of diagnosis and management [2]. Early recognition is critical, as prompt initiation of high-dose glucocorticoid (GC) and immunosuppressive therapy has been associated with improved neurological outcomes in many cases [3]. However, SLE-associated myelitis can mimic other spinal disorders, particularly demyelinating diseases such as neuromyelitis optica spectrum disorder (NMOSD) or multiple sclerosis, and even intramedullary tumors, including spinal glioblastoma [4]. Spinal glioblastoma represents a particularly high-risk differential diagnosis because of its aggressive nature and the frequent need for early surgical intervention. Inflammatory myelitis has been reported to radiologically resemble spinal cord tumors, leading in some instances to diagnostic delay or unnecessary invasive procedures. Conversely, cases of spinal glioblastoma initially treated as autoimmune myelitis have also been described, underscoring the diagnostic overlap between inflammatory and neoplastic conditions [5]. On MRI, intramedullary T2 hyperintensity with variable or heterogeneous gadolinium enhancement may contribute to this diagnostic confusion. Therefore, a comprehensive evaluation, including magnetic resonance imaging (MRI) findings, cerebrospinal fluid (CSF) analysis, serologic testing, and assessment of GC responsiveness, is essential.

Here, we report a case of SLE-associated myelitis that radiologically mimicked spinal glioblastoma and was initially difficult to differentiate from an intramedullary tumor. This case suggests the importance of recognizing that SLE-associated myelitis may closely mimic intramedullary tumors such as spinal glioblastoma, and indicates that GC responsiveness could serve as a useful diagnostic clue in distinguishing inflammatory from neoplastic conditions.

## Case presentation

A 48-year-old woman had been diagnosed with SLE 17 years earlier on the basis of rash, non-erosive arthritis, leukopenia, high-titer antinuclear antibody (ANA) positivity (1:1280, homogeneous/speckled), hypocomplementemia, and anti-double-stranded DNA (dsDNA) immunoglobulin G (IgG) positivity. She had been under regular follow-up in our rheumatology division. Approximately three weeks before admission, she developed numbness and weakness radiating from the lumbar region to the bilateral lateral thighs, particularly triggered by cervical flexion. Two weeks before admission, she visited the orthopedic department, where spinal MRI suggested an intramedullary tumor, and she was subsequently referred and admitted to our department for further evaluation. On admission, she was alert and fully oriented. Extraocular movements were intact, and facial sensory and motor function were normal. No dysarthria was noted, and the tongue protruded midline. Both the Barre and Mingazzini tests were negative. Manual muscle testing revealed full strength in the upper extremities (5/5), and mildly reduced left iliopsoas strength (5/5-), with all other lower limb muscles graded 5/5. Deep tendon reflexes, including biceps, brachioradialis, triceps, patellar, and Achilles reflexes, were normal bilaterally. No pathological reflexes were observed (Babinski, Chaddock, Hoffmann, and Tromner all negative). Sensory examination revealed mild hypoesthesia over both lateral thighs without laterality. No ataxia was observed, gait was stable, and bladder and bowel function were preserved.

Laboratory findings showed no evidence of significant inflammatory response or hematologic abnormality, and renal and hepatic function tests were within normal limits (Table 1).

**Table 1:** Laboratory and cerebrospinal fluid findings. ALT, alanine aminotransferase; anti-dsDNA IgG, anti-double-stranded DNA immunoglobulin G; anti-ssDNA IgG, anti-single-stranded DNA immunoglobulin G; AST, aspartate aminotransferase; CH50, 50% hemolytic complement activity; CSF, cerebrospinal fluid; MOG antibody, myelin oligodendrocyte glycoprotein antibody; sIL-2R, soluble interleukin-2 receptor

Category	Parameter	Result	Reference Range
Hematology	White blood cell count	4,900 /µL	3,500-9,000 /µL
	Hemoglobin	11.8 g/dL	11.5-15.0 g/dL
	Platelet count	327,000 /µL	150,000-400,000 /µL
Inflammation	C-reactive protein	0.2 mg/dL	<0.3 mg/dL
Renal function	Serum creatinine	0.77 mg/dL	0.46-0.79 mg/dL
Hepatic function	AST	18 U/L	13-30 U/L
	ALT	7 U/L	7-23 U/L
Complement	C3	131 mg/dL	73-138 mg/dL
	C4	13 mg/dL	11-31 mg/dL
	CH50	42.1 U/mL	30-45 U/mL
Autoimmune serology	Anti-dsDNA IgG	13 IU/mL	<12 IU/mL
	Anti-ssDNA IgG	28 AU/mL	<25 AU/mL
	Anti-Smith antibody	1.3 U/mL	<7.0 U/mL
	Aquaporin-4 antibody	<1.5	<1.5
	MOG antibody	<1.5	<1.5
CSF	Cell count	9 /µL	≤5 /µL
	Cell differential (monocytes/PMNs)	9 / 0	Monocytes predominant
	Protein	32 mg/dL	10-40 mg/dL
	Glucose	59 mg/dL	45-80 mg/dL
	Oligoclonal bands	Negative	Negative
CSF/serum	CSF IgG	4.0 mg/dL	0.3-4.0 mg/dL
	CSF albumin	19 mg/dL	5-35 mg/dL
Serum	Serum IgG	2195 mg/dL	870-1700 mg/dL
	Serum albumin	4.0 g/dL	3.8-5.3 g/dL
	sIL-2R	317 U/mL	121-613 U/mL
Calculated	IgG index	0.38	<0.7

Complement levels were preserved, and autoimmune serology was consistent with her known diagnosis of SLE. Both aquaporin-4 and myelin oligodendrocyte glycoprotein antibodies were negative, supporting exclusion of NMOSD. CSF analysis revealed mild pleocytosis without oligoclonal bands, while protein and glucose levels were within normal ranges. The immunoglobulin G (IgG) index was not elevated, indicating the absence of intrathecal IgG synthesis.

**Figure 1:**
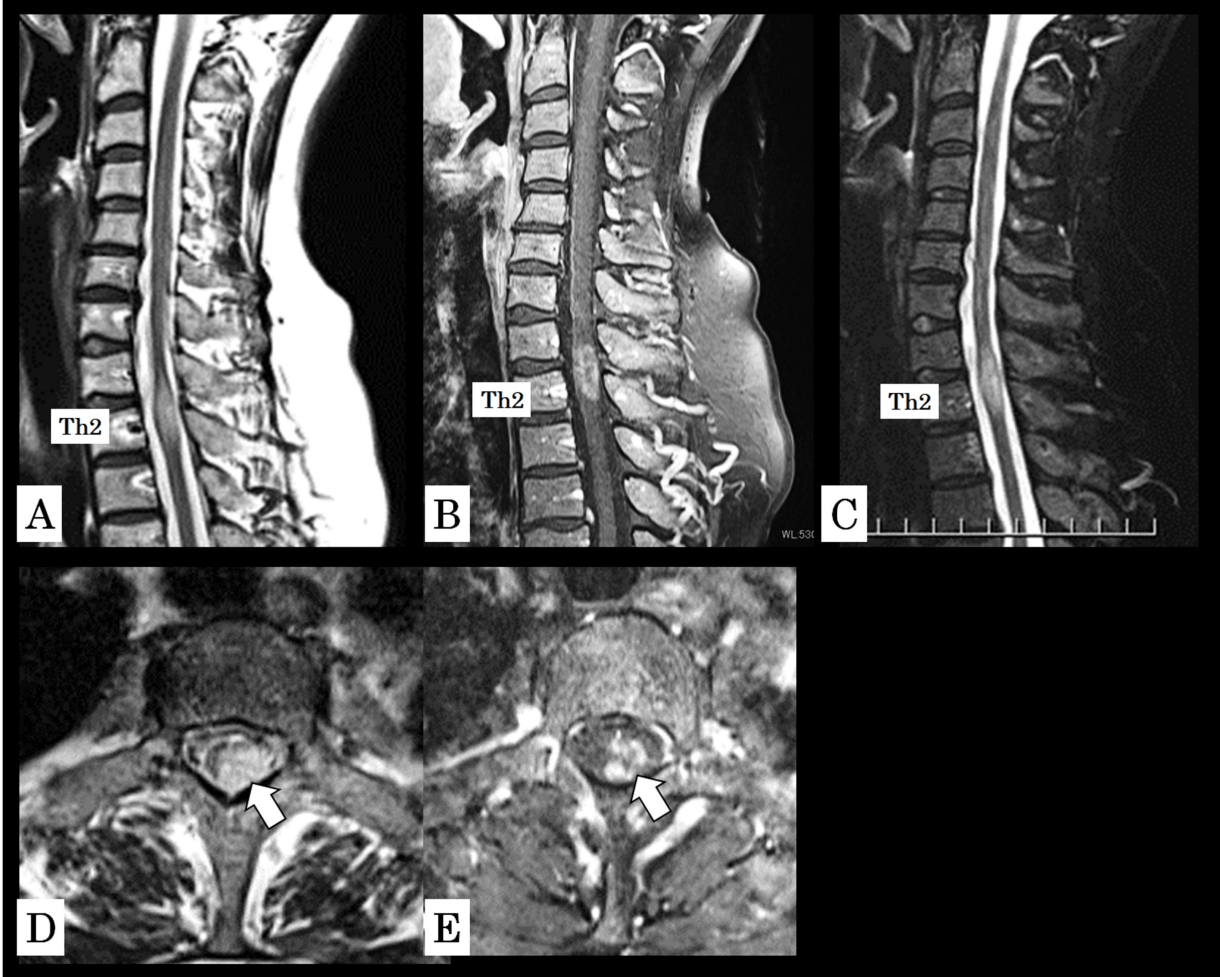
Cervical spine MRI findings. (A) T2-weighted sagittal image showing an intramedullary hyperintense lesion at Th1-2. (B) Gadolinium-enhanced sagittal image demonstrating a relatively well-demarcated intramedullary mass with mildly heterogeneous enhancement. (C) Fat-suppressed sagittal image in which the intramedullary high signal appears more conspicuous, with accentuated contrast against the surrounding spinal cord. (D) Axial T2-weighted image and (E) axial gadolinium-enhanced image showing the lesion at the corresponding level (arrows indicate the lesion).

CSF cultures and polymerase chain reaction testing were negative, and FilmArray meningitis/encephalitis panel revealed no pathogens. Spinal MRI demonstrated an intramedullary lesion at Th1-2 that appeared hyperintense on T2-weighted images. On gadolinium-enhanced images, the lesion was visualized as a relatively well-demarcated intramedullary mass with mildly heterogeneous enhancement, raising suspicion for an intramedullary tumor, including spinal glioblastoma. On fat-suppressed images, the intramedullary high signal became more conspicuous, with accentuated contrast between the lesion and the surrounding spinal cord (Figures 1A-1E).

Brain MRI showed no abnormalities.

Although surgical intervention was initially considered, SLE-associated myelitis was also suspected. Therefore, GC therapy was initiated as a diagnostic and therapeutic trial. Dexamethasone was selected because it provides potent anti-inflammatory effects for acute spinal cord inflammation and edema and is commonly used in neurosurgical practice when both inflammatory and neoplastic etiologies are being considered. Importantly, because an intramedullary tumor could not be completely excluded at presentation, a relatively low initial dose was intentionally chosen to allow assessment of treatment responsiveness while minimizing the risk of masking an underlying neoplastic process.

The starting dose of dexamethasone was 4 mg/day, corresponding to approximately 0.06 mg/kg/day based on the patient’s body weight of 65 kg. This dose was considered sufficient for a diagnostic steroid trial while allowing close neurological monitoring.

**Figure 2:**
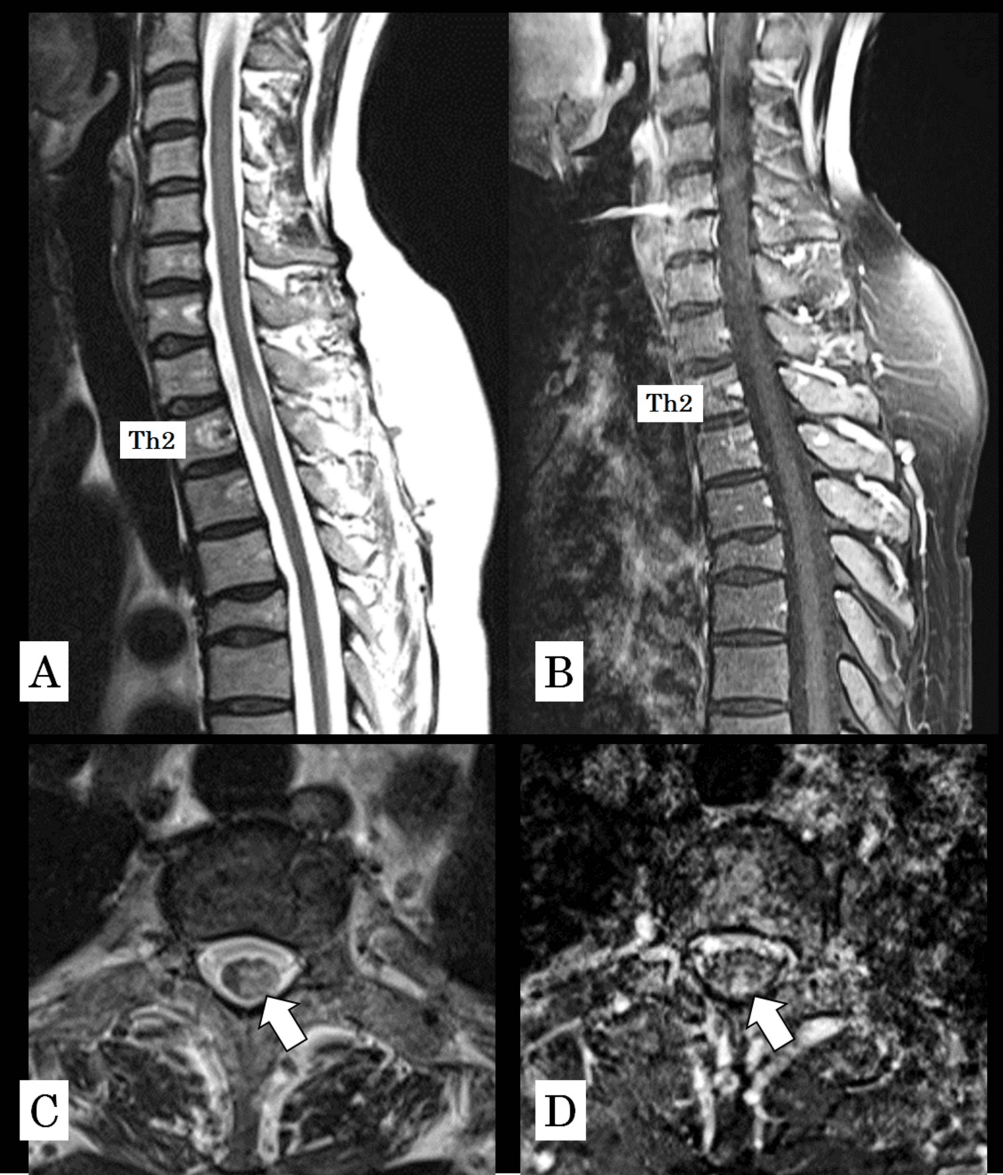
Thoracic spine MRI on day 7 of treatment. (A) T2-weighted sagittal image showing improvement in T2 hyperintensity and reduction in lesion size. (B) Gadolinium-enhanced sagittal image demonstrating decreased contrast enhancement compared to initial imaging. (C) Axial T2-weighted image and (D) axial gadolinium-enhanced image confirming reduction in lesion extent (arrows indicate the lesion).

The patient’s sensory symptoms improved within several days, and a follow-up MRI performed on day 7 demonstrated improvement in T2 hyperintensity, reduction in lesion size, and decreased contrast enhancement (Figures 2A-2D).

**Figure 3:**
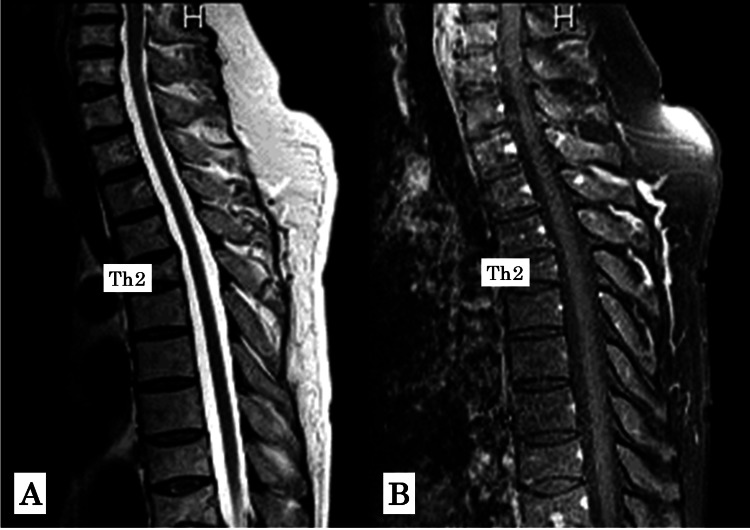
Thoracic spine MRI at three months after treatment initiation. (A) T2-weighted sagittal image showing normalization of signal intensity. (B) Gadolinium-enhanced sagittal image demonstrating resolution of contrast enhancement.

Based on these clinical and radiological findings, SLE-associated myelitis was considered the most likely diagnosis, and surgical intervention was deferred in favor of conservative management. Dexamethasone was gradually tapered in the outpatient setting over several weeks. Follow-up MRI at three months demonstrated normalization of both T2-weighted and contrast-enhanced images (Figures 3A-3B).

At the 20-month follow-up, the patient remained relapse-free with a stable neurological condition.

## Discussion

Spinal intramedullary lesions pose a diagnostic challenge, as they encompass a wide range of etiologies, including inflammatory demyelination, autoimmune myelitis, infection, and neoplasms such as spinal glioma or glioblastoma. The absence of oligoclonal bands and a normal IgG index supported exclusion of demyelinating disease. Although the rapid response to GC therapy favored an inflammatory etiology, treatment response alone is not diagnostic. Given the mild neurological deficits and the risk associated with intramedullary biopsy, further invasive procedures were not pursued.

In the present case, the lesion demonstrated T2 hyperintensity and heterogeneous gadolinium enhancement on MRI, initially raising suspicion of an intramedullary tumor, particularly spinal glioblastoma. Among neoplastic differential diagnoses, spinal glioblastoma was considered the most likely based on both imaging characteristics and clinical context. Metastatic intramedullary tumors were considered less probable because the patient had no history of systemic malignancy, and a comprehensive evaluation, including a brain MRI, revealed no evidence of a primary tumor or additional metastatic lesions. Intramedullary metastases are typically associated with known systemic cancer and often present as multiple lesions, which was inconsistent with the present case. Primary spinal malignant lymphoma was also considered; however, lymphoma frequently demonstrates relatively homogeneous and intense gadolinium enhancement, marked spinal cord enlargement, or leptomeningeal involvement. In contrast, the lesion in this case showed mildly heterogeneous enhancement without diffuse cord swelling or meningeal involvement, and CSF analysis revealed no malignant cells or significant protein elevation.

Spinal glioblastoma, although rare, is known to present as a solitary intramedullary lesion with heterogeneous enhancement and mass-like appearance, occasionally mimicking inflammatory lesions. Given the focal, tumor-like intramedullary morphology at the Th1-2 level and the absence of systemic malignancy, spinal glioblastoma was considered the most plausible neoplastic diagnosis prior to assessment of treatment responsiveness.

Spinal glioblastoma is rare but typically presents with progressive neurological decline and often requires surgical biopsy or resection for diagnosis [6]. However, radiological differentiation between neoplastic and inflammatory lesions is not always straightforward, and misdiagnosis may lead to unnecessary surgical intervention. Radiologically, spinal glioblastoma and SLE-associated myelitis share several overlapping features, which may contribute to diagnostic difficulty. Both can appear as intramedullary lesions with T2 hyperintensity and variable gadolinium enhancement [6]. Patchy or ring-like enhancement observed in inflammatory myelitis may resemble neoplastic neoangiogenesis, and spinal cord swelling can be present in both conditions [7]. These similarities highlight the limitations of single-time-point MRI in distinguishing inflammatory from neoplastic pathology. Certain radiological characteristics, however, may assist in differentiating the two. Spinal glioblastoma often demonstrates progressive enlargement, pronounced mass effect, and heterogeneous enhancement with necrotic or cystic components as the disease advances [6]. In contrast, SLE-associated myelitis may present with diffuse or longitudinally extensive involvement without a discrete nodular mass, and contrast enhancement is generally less aggressive [8].

Serial MRI evaluation can be valuable, as inflammatory lesions typically improve after GC therapy, whereas glioblastoma tends to progress despite medical management [9]. In the present case, marked radiological improvement within seven days of steroid initiation supported an inflammatory rather than neoplastic etiology. Primary spinal malignant lymphoma was considered in the differential diagnosis; however, it was deemed less likely because the patient had no systemic symptoms, no cytopenias or inflammatory abnormalities, and a normal serum soluble interleukin-2 receptor level (317 U/mL). Notably, the gadolinium-enhanced images demonstrated a relatively well-demarcated intramedullary mass with mildly heterogeneous enhancement, a finding that could mimic an intramedullary tumor. The hyperintensity became more conspicuous on fat-suppressed sequences, further sharpening the contrast between the lesion and the surrounding spinal cord. Although these features were suggestive of a neoplastic lesion, the rapid reduction of enhancement and subsequent normalization on follow-up MRI supported an inflammatory etiology rather than a tumor.

SLE-associated myelitis is a rare but clinically important neurological manifestation, occurring in fewer than 3% of SLE patients [10]. It may present acutely and lead to irreversible deficits if treatment is delayed. Because MRI findings may mimic intramedullary tumors, diagnosis may be challenging, underscoring the importance of integrating clinical context, serological data, CSF analysis, and therapeutic response. In this case, the absence of oligoclonal bands, normal IgG index, and negative infectious testing reduced the likelihood of multiple sclerosis or infection. The rapid symptomatic and radiological improvement following GC administration was a key factor supporting inflammatory myelitis and avoiding unnecessary surgery. The patient remained relapse-free for 20 months under a tapering GC regimen, suggesting a monophasic inflammatory course. This case emphasizes the need to consider autoimmune myelitis when evaluating intramedullary spinal lesions and suggests that GC responsiveness, together with serial MRI assessment, may serve as a practical diagnostic approach when tumor remains a differential concern.

This report has several limitations. First, the diagnosis of SLE-associated myelitis was made clinically based on imaging, serological background, exclusion of infection, and favorable GC response, without histopathological confirmation. Therefore, alternative inflammatory etiologies cannot be completely excluded. Second, as a single case report, the findings may not be generalizable to all patients with autoimmune myelitis or intramedullary tumors. Third, although early radiologic improvement was observed, long-term disease stability should ideally be confirmed with continued MRI follow-up. Finally, the optimal immunosuppressive strategy and the role of biopsy in diagnostically ambiguous cases remain areas for future investigation.

## Conclusions

SLE-associated myelitis can closely mimic intramedullary spinal tumors such as spinal glioblastoma on MRI, posing a significant diagnostic challenge. In this case, careful consideration of the clinical background and CSF findings, together with short-interval follow-up MRI, was essential to avoid unnecessary surgical intervention. The rapid clinical and radiological response to GC therapy strongly supported an inflammatory rather than neoplastic etiology. This case highlights the importance of considering autoimmune myelitis in the differential diagnosis of intramedullary spinal cord lesions and suggests that GC responsiveness and serial imaging are valuable tools for accurate diagnosis and appropriate management.
